# Epidemiology and National Surveillance System for Foot and Mouth Disease in Cattle in Thailand during 2008–2019

**DOI:** 10.3390/vetsci7030099

**Published:** 2020-07-24

**Authors:** Orapun Arjkumpa, Tedsak Yano, Rotchana Prakotcheo, Chalutwan Sansamur, Veerasak Punyapornwithaya

**Affiliations:** 1 Ph.D. Degree Program in Veterinary Science, Faculty of Veterinary Medicine, Chiang Mai University, Chiang Mai 50100, Thailand; orapun_arj@cmu.ac.th; 2 Department of Food Animal Clinic, Faculty of Veterinary Medicine, Chiang Mai University, Chiang Mai 50100, Thailand; tedsak.yano@cmu.ac.th; 3 Bureau of Disease Control and Veterinary Services, Department of Livestock Development, Bangkok 10400, Thailand; dr_sam339@hotmail.com; 4 Akkhraratchakumari Veterinary College, Walailak University, Nakhon Si Thammarat 80161, Thailand; chakream@gmail.com; 5 Veterinary Public Health Centre for Asia Pacific, Faculty of Veterinary Medicine, Chiang Mai University, Chiang Mai 50100, Thailand

**Keywords:** surveillance, foot and mouth disease, cattle, Thailand

## Abstract

Foot and mouth disease (FMD) is a prominent transboundary disease that threatens livestock production and can disrupt the trade in animals and animal products at both regional and international levels. The aims of this study were: (1) to analyze the distribution of FMD in Thailand during the period of 2008 to 2019, (2) to outline a national surveillance approach, and (3) to identify the existing knowledge gap that is associated with this disease in relation to cattle production. We analyzed FMD outbreak data in order to determine the existing spatial and temporal trends and reviewed relevant publications and official documents that helped us outline a national surveillance program. There were 1209 FMD outbreaks in cattle farms during the study period. FMD outbreaks occurred every year throughout the study period in several regions. Notably, FMD serotype O and A were considered the predominant types. The FMD National Strategic Plan (2008–2015) and the national FMD control program (2016–2023) have been implemented in order to control this disease. The surveillance approach employed by livestock authorities included both active and passive surveillance techniques. The vaccination program was applied to herds of cattle 2–3 times per year. Additionally, numerous control measures have been implemented across the country. We have identified the need for a study on the assessment of an applicable surveillance program, the evaluation of an appropriate vaccination strategy and an assessment of the effectiveness of a measured control policy. In conclusion, this study provided much needed knowledge on the epidemiology of FMD outbreaks across Thailand from 2008 to 2019. Additionally, we identified the need for future studies to address the existing knowledge gaps. The findings from this study may also be useful for livestock authorities and stakeholders to establish an enhanced control strategy and to implement an effective surveillance system that would control and eradicate FMD throughout the country.

## 1. Introduction

Foot and mouth disease (FMD) is a prominent transboundary disease among animals that can seriously impact livestock production and disrupt the trade in animals and animal products at both the regional and international levels [1]. It is caused by a virus of the genus Aphthovirus, family *Picornaviridae*. Seven serotypes have been known to infect cloven-hoofed animals: O, A, C, SAT 1, SAT 2, SAT 3, and Asia-1. Importantly, each serotype infection does not grant immunity to another infection [1]. Over the last 40 years, different genetic subtypes of this disease have persisted in Thailand within a limited range of the bordering countries. However, the disease is known to spread periodically and temporarily beyond these boundaries [2,3]. Recently, six serotypes of the FMD virus (O, A, SAT 1, SAT 2, SAT 3, and Asia-1) have been circulating globally [4] and remain a persistent threat of incursions in large parts of Africa, the Middle East, and Asia [1,5].

For the development of an effective FMD control program, several regional challenges persist, including uncontrolled animal movements, difficulties in supplying vaccines and establishing their efficacy, limited technical skills in the field, insufficient biosecurity networks, low levels of involvement of local disease control programs, and the inherent complexities of integrating national and international control programs [6]. However, an universal strategy for FMD control was endorsed in 2012 at the global level [7]. The Food and Agriculture Organization of the United Nations (FAO) and the World Organization for Animal Health (OIE) launched the Progressive Control Pathway for FMD control (PCP-FMD) with the goal of eradicating FMD worldwide in 2014 [8]. The initial measures outlined in this strategy include early detection and alert systems and the implementation of successful surveillance programs, as has been stated in the OIE Terrestrial Code guidelinesThe global FMD control strategies include improving global FMD control by the enhancement of field and laboratory technical capabilities and the expansion and implementation of effective disease control measures. These measures would include vaccines, surveillance, biosecurity, compliance with movement regulations and public awareness campaigns to regulate FMD, strengthening veterinary services, as well as improving the prevention and control of other major diseases known to affect cattle [6,7].

Disease surveillance is an important epidemiological tool used to monitor the health of a population. The goals of an effective disease surveillance program are mainly to describe the current burden and epidemiology of the disease, to monitor current trends and to identify outbreaks and new pathogens [9]. Examples of successful FMD eradication programs that have been implemented in some countries of Southeast Asia, which include effective surveillance programs, have revealed that intensive epidemiological surveillance can be undertaken to track the potential reappearance of FMD cases. One of these successful programs was utilized in Indonesia [10]. Additionally, the Philippines established a method to strengthen their disease monitoring and surveillance activities in order to eliminate cases of the disease that reemerged in high risk areas during implementation of an FMD eradication program [11].

Thailand has been recognized as an endemic area of FMD for more than 60 years [12]. FMD serotype O and A are common serotypes that are found in this country [13,14]; however, serotype Asia-1 FMD has not been observed since 1998 [12]. Cattle, buffalo, and pigs were identified as the main FMD affected species during the outbreak over the previous decades [13,15,16]. To effectively control FMD and eradicate it from the country, the FMD National Strategic Plan of 2008–2015 [17] and the national FMD control program of 2016–2023 [18] were implemented by the Department of Livestock Development of Thailand (DLD).

The aims of this study were: (1) to analyze the distribution of FMD in Thailand from 2008 to 2019; (2) to describe a national surveillance approach; and (3) to identify the existing knowledge gap with regard to the disease in terms of cattle production.

## 2. Materials and Methods

### 2.1. Data Sources and Outbreak Definitions

FMD outbreak and surveillance data for cattle were obtained from DLD and OIE between 2008 and 2019 [19]. All of the acquired data were used in this study. Additionally, we used Google Scholar and Google search tools to retrieve publications that are related to FMD surveillance using English and Thai keywords in order to find the relevant literature published in national and international journals during the years from 1993 to 2020. In addition, we intensively explored the main DLD and DLD subdivision websites, such as those belonging to the Bureau of Disease Control and Veterinary Services (BDCVS), the Regional Reference Laboratory for Foot and Mouth Disease in South East Asia (RRLSEA), and the Regional Livestock Offices (RLO) including Region 1 to 9, in order to obtain FMD information from official documents, reports, and research publications available online. Additionally, data were collected through direct contact with responsible persons from the DLD and DLD subdivisions.

Data including location (provincial level), date, and time of outbreak onset, along with the serotype of the FMD virus for each outbreak, were used to create a series of maps that represent the spatial and temporal distribution of FMD.

An outbreak is characterized as a cattle farm in which at least one animal displayed the typical clinical signs of FMD, including vesicles on the feet, mammary glands, and around the oral cavity. For some outbreak farms, tissue samples (e.g., oral epithelium and vesicle lesion tissue) or blood samples taken from some clinical FMD cattle specimens were collected and were confirmed as being FMDV positive using the PCR method or ELISA technique, respectively [20]. The laboratory testing was performed at the Regional Veterinary Research and Development Centers (VRDCs) and/or RRLSEA in Thailand.

### 2.2. Data Analysis

Monthly distributions of FMD outbreaks by serotype between the years of 2008 and 2019 were analyzed using R statistical software version 3.6.3 (R Core Team, Vienna, Austria) [21] using EpiCurve function [22]. A series of maps for FMD outbreaks were created using QGIS version 2.18.28 (Open Source Geospatial Foundation Project, Zurich, Switzerland) [23]. Poisson regression analysis in R was used to compare the number of FMD outbreaks that occurred during the periods of 2008–2013 and 2014–2019. Accordingly, the FMD national strategic plan, surveillance systems, control measures, sero-surveillance survey, nucleotide sequencing, and epidemiological studies have all been reviewed.

## 3. Results

Reports and publications used in the study were divided by five topics including distribution of FMD outbreaks (*n* = 8), national strategic plan (*n* = 6), FMD surveillance, vaccine and control measures (*n* = 7), sero-surveillance survey (*n* = 5), and FMD epidemiological studies (*n* = 22).

### 3.1. FMD Outbreak

**Figure 1:**
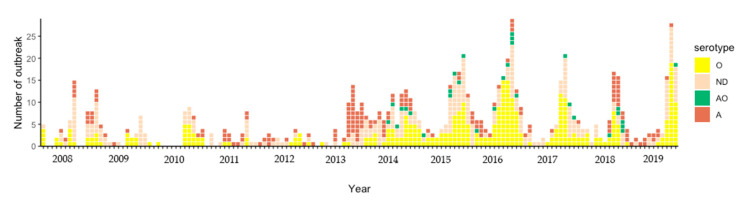
Monthly distribution of foot and mouth disease (FMD) outbreaks among cattle in Thailand by serotypes from 2008 to 2019; O = serotype O, A = serotype A, AO = serotype A and O, and ND = not identified, not sampled, and pending.

**Figure 2:**
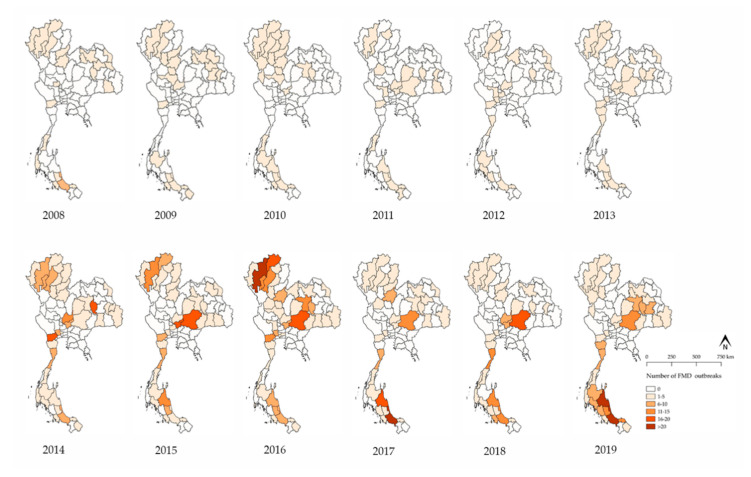
Yearly distribution of FMD outbreaks among cattle in Thailand by province from 2008 to 2019.

**Figure 3:**
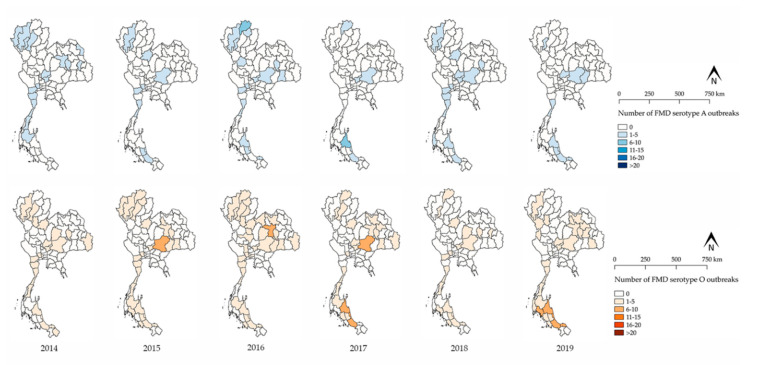
Yearly distribution of FMD outbreaks by serotype (blue color = serotype A, orange color = serotype O) among cattle in Thailand by province from 2014 to 2019.

There were 1209 FMD outbreaks in cattle farms over the course of the study period. Additionally, FMD outbreaks among cattle were reported each and every year during the study period (Figure 1). Incidences of FMD outbreaks have been increasing in the last six years. The most massive FMD outbreak occurred during the year of 2015–2016. The major FMD serotypes were found to be serotype O and then serotype A. Since 2014, data have shown that serotype O was the predominant serotype in all FMD outbreaks. Notably, there have been no reports of Asia-1 during this study period. However, FMD outbreaks have been reported in the following three FMD areas in Thailand: (1) the north-central-west, (2) the north-east, and (3) the southern regions with the exception of the eastern region (Figure 2). From 2014 to 2016, the northern region experienced the highest number of FMD outbreaks, especially in 2016. The spatial distribution of FMD serotype A and O was shown in Figure 3. Furthermore, the number of FMD outbreaks in the southern region increased over the last three years, whereas the central region experienced an almost consistent number of FMD outbreaks from 2014 to 2019. The results from the Poisson model (Table 1) indicated that the number of outbreaks during the period of 2014 to 2019 was higher than the period of 2008 to 2013 (risk ratio = 1.69; 95% CI = 1.28–2.22).

### 3.2. FMD National Strategic Plan

**Figure 4:**
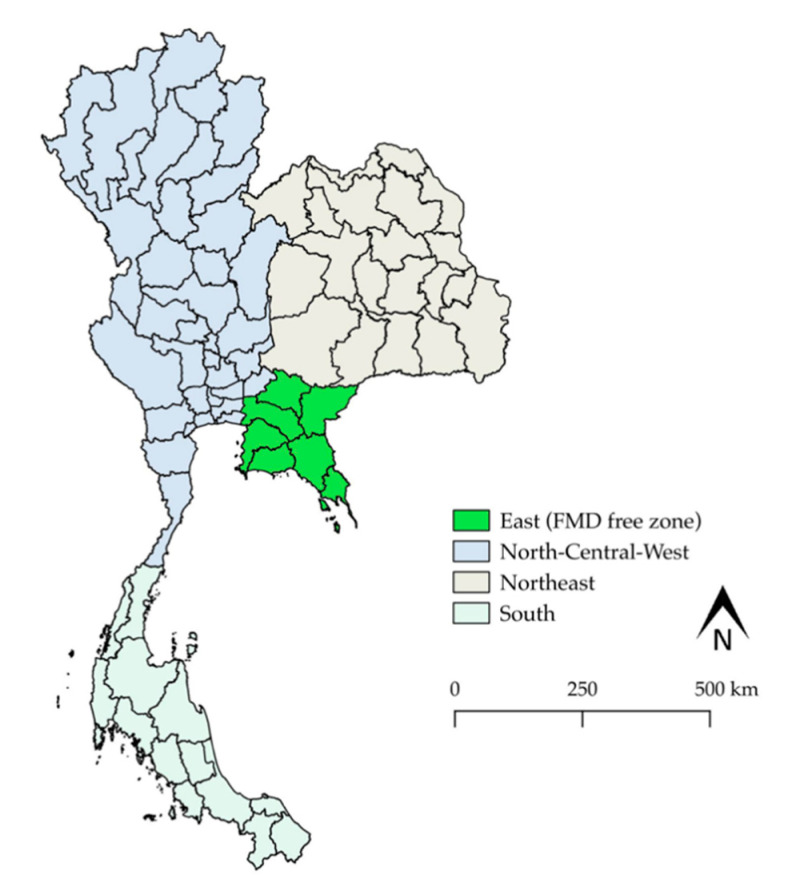
FMD zoning in Thailand.

The DLD in Thailand has developed an FMD National Strategic Plan (2008–2015) consisting of a broad range of activities that are in harmony with the Southeast Asia Foot and Mouth Disease campaign. The details of this plan have been well described by DLD and Yano et al. [17,24]. In brief, this plan provides a long-term strategic framework along with guidance on how to accomplish the eradication of FMD in Thailand. The main objectives of the plan were to reduce the risk of FMD infections and the spread of the disease, as well as to increase the capacity to detect FMD through rapid control and eradication actions. In addition, Thailand has been committed to establishing FMD-free areas throughout four FMD zones that have been set up to explicitly monitor the disease in terms of its numerous epidemiological causes. All of this will contribute to the complete eradication of FMD in the country. Notably, the eastern part of Thailand was the first to create an FMD-free zone (Figure 4) [13]. The PCP-FMD stage for Thailand was in between stage 3 and stage 4 for the eastern region, and stage 3 for other regions [25]. Recently, the national FMD control program for 2016–2023 was submitted to the OIE for endorsement with the aim of allowing Thailand to become a country that is completely free of FMD by 2023 [13,18]. This protocol would be applied in the hopes of declaring Thailand an FMD-free country [26].

### 3.3. Structure of FMD Surveillance by DLD Authority

Surveillance approaches for effective FMD eradication among cattle in Thailand have combined both active and passive surveillance techniques [27]. The core components of effective FMD active surveillance approaches include active clinical surveillance and active specific surveillance techniques. For the active surveillance technique, active clinical (case detection) surveillance is implemented as it aims to notify local veterinary officers about an FMD outbreak. Since there are routine visits at cattle farms once a month by livestock officers, if any FMD-like symptoms appear among the cattle herds, the authority will immediately inform the District Livestock Office and Provincial Livestock Office in order to initiate a deeper investigation by collecting samples and then send them to the laboratory for virus detection. Specific surveillance is performed by BDCVS. This surveillance program involves monitoring the status of FMD for imported cattle into the country and also involves monitoring the status of FMD among cattle moving within the country from FMD zones to FMD-free zones. Additionally, sero-surveillance techniques were also performed every year in some areas to identify antibody titers among both dairy and beef cattle following the administration of mass vaccinations. For passive surveillance programs, farmers or livestock volunteers in the affected area are expected to be on the alert for clinical signs of the disease and to report these signs to local livestock officers as quickly as possible. Veterinary officers would then input any suspected cases of FMD into the reporting system in the form of both paper and electronic data. For laboratory surveillance, the National Institute of Animal Health, VRDCs, and RRLSEA are all responsible for conducting routine laboratory surveillance and monitoring for FMD.

### 3.4. FMD Vaccination and Control Measures

Since 2008, the FMD vaccination campaign with more than 80% coverage nationwide has been applied two and three times yearly for beef and dairy cattle, respectively. A ring vaccination was utilized during the outbreak period [17,18]. Trivalent (serotypes O, A, and Asia-1), bivalent (serotypes O and A), and monovalent (serotype O) vaccines that were produced by the DLD have been used in different areas depending on the serotype of each outbreak. Currently, FMD lineages that include serotype A/Lopburi/2012, serotype O/189/87, and serotype Asia-1/Thailand/85 were used in the vaccines [28,29]. Several studies have indicated that vaccine matching was acceptable as the serological relationship between field isolate viruses and the reference virus. Consequently, vaccine strain values (r-value) from most of the samples (>80%) were greater than 0.40 [28,29]. In general, vaccinations were administered by trained farmers, veterinary-para professionals, and veterinarians. For the control measures, numerous efforts were made to implement an effective FMD National Strategic Plan across the country that included restrictions on cattle movements and enhancement of biosecurity measures on farms and in surrounding areas [13,24].

### 3.5. Seroprevalence Survey of FMD and Nucleotide Sequencing

Sero-surveillance surveys have been performed every year, which aimed to detect infected animals and determine the protective level of antibody titers of vaccinated animals using the nonstructural protein (NSP) test and liquid phase blocking (LP) ELISA, respectively [17,29,30]. A recent report showed that the FMD serotype O/189/87, serotype A/Lopburi/2012, and serotype A/Sakolnakorn/97 were the predominant FMD serotypes in 2019 [29]. Additionally, serotype O with Mya-98/SEA, Ind-2001e/ME-SA, Pan Asia/ME-SA, and serotype A with Sea-97/ASIA were also reported [29].

### 3.6. Epidemiological Study for FMD

In recent decades, FMD studies have been focused on the specific outbreak characteristics and patterns [31,32,33,34,35,36,37,38], risk factors [32,34,39,40,41,42,43], risk assessment [44,45], knowledge, attitude, and practice (KAP) surveys [46,47], network analysis [41], spatiotemporal analysis [20,40], spatial analysis [48], vaccines [28], diagnostic technique development [49,50], serological analysis [30,51,52,53,54], and the relevant control measures [24,25,55].

## 4. Discussion

Although the national strategic plan and control measures have been implemented, FMD still remains a critical problem for cattle farmers in Thailand. Several important points and the existing knowledge gaps regarding FMD are discussed in this study.

The number of FMD outbreaks has increased in the last six years (Table 1). Both FMD serotype O and A were spatially distributed across Thailand (Figure 3). This finding may reflect the true outbreak situation, wherein more outbreaks may have occurred or, with high certainty, this upward trend may be the result of an enhancement in the reporting system implemented. As the new system supports both passive and active surveillance systems, more reports of outbreaks would be expected. Most outbreaks occurred in the northern region from 2014 to 2016, but after 2016, a high number of outbreaks were reported in the southern region (Figure 2). Since the massive outbreak that occurred in northern Thailand in 2015–2016, the DLD has implemented a rigid set of FMD control measures [24], which could lead to a decrease in the number of outbreaks. Specifically, FMD serotype O was the predominant type causing numerous outbreaks (Figure 1). This finding raises a question for future studies as to why the FMD serotype O has been the main cause of FMD for a long period of time in Thailand and what factors contribute to the spread of the FMD virus.

Evaluation of the surveillance programs is a critical component for the success of any system [56,57,58,59,60]. Appropriate surveillance assessment plays a vital role in the establishment and maintenance of international confidence [61]. Based on this study and our knowledge, there has been no country-wide evaluation of FMD surveillance in Thailand. Thus, the evaluation of the national FMD surveillance system should be set as a priority.

As the FMD outbreak is highly contagious and the spread of the disease can occur via direct and indirect contact [62], early detection and early warning systems are essential for the investigation of FMD outbreaks and the control of the disease. For case detection, we have recommended that the education of cattle farmers is strictly needed. Prompt responses from local veterinary officers would be a remarkable component of enhancing an early detection system. In addition, the outbreak investigation program should then be carried out by well-trained staff. A systematic and organized investigation of the outbreak will help stakeholders to understand the outbreak characteristics and identify potential risk factors of an outbreak [63]. The implementation of a risk-based surveillance system should be strengthened and enhanced, as it can be very supportive of an active surveillance system. Moreover, participatory livestock disease surveillance should be implemented to develop better animal health programs and more successful surveillance and control strategies [64].

Vaccinations are the primary preventive measure in protecting cattle against FMD. While vaccinations are typically conducted 2 to 3 times a year, FMD outbreaks still occur in areas where vaccinations have been administered [39]. The following practices could be a possible reason for unsuccessful vaccination programs. First, there is the tendency for some animals to be exempt from receiving vaccinations (e.g., pregnant animals, animals sent out for grazing at a distance, and sick animals) as was found in Sri Lanka [65]. Second, if vaccinations were administered by less experienced farmers, it is possible that the handling of the vaccine and vaccination techniques may not be appropriate. Third, inadequate storage of vaccines may also reduce the efficacy of the vaccines. Last, there is a chance that the spread of the FMD virus could have occurred before the vaccine protective titer was achieved. Thus, practical training for the vaccinator would be necessary. Future research on an effective vaccination policy needs to be carried out to provide information on strengthening the current vaccination program.

A better understanding of the epidemiology of FMD will support an effective surveillance system. Most epidemiological studies report important epidemiological information such as prevalence and risk factors of FMD outbreaks that are useful for formulating control strategies but more future studies are needed. For instance, several studies have indicated that the movement of new cattle into farms without quarantine is the significant risk factor for FMD outbreaks [39,40,42]. However, a recent study found that most of the farmers in FMD endemic areas do not conduct such quarantine [39]. Thus, it is interesting to determine whether the current control strategies are well-adopted by farmers since this practice has been implemented through 2008–2015 national strategic plan. Moreover, it is important to determine the effectiveness of the national control strategies in the future study, as it will provide essential knowledge to strengthen the existing control strategies. Additionally, since the application of advanced epidemiological methods such as spatial statistics and network analysis to epidemiological studies with provincial datasets [20,40,41,48] provides a better understanding of FMD epidemiology, such methods should be applied for the analysis of larger datasets, such as regional and national data for future studies.

The relevant data related to the FMD situation in Thailand could be useful for future studies that will focus on improving an FMD surveillance system and the associated control measures. More epidemiological studies using other advanced methods are needed. Similar to other countries [66,67], one of the circumstances of concern is the under-reporting of incidences of FMD, which can result in an underestimation of the true disease situation [68]. With the capture-recapture method using two or more data sources [69,70,71], the true status of FMD could be estimated. Moreover, a hierarchical Bayesian model should be used to create a spatial risk map that would be very useful for risk communication [72]. Additionally, the application of mathematical models for disease transmission and control could provide information relevant to the policy decision-making process. Furthermore, we have determined that it would be imperative to conduct more studies on measuring the subclinical infection status of cattle. This could impact FMD transmission along with field vaccine trial studies implemented to determine the efficiency of vaccines and an effective vaccination strategy.

## 5. Conclusions

This was the first study that accurately describes the FMD status and relevant surveillance approaches among cattle at the national level and identifies certain gaps that could help the development of effective surveillance systems in Thailand. Although the national FMD strategic plan and the national FMD control program have been implemented, FMD outbreaks have still occurred in some regions. Consequently, we have identified the need for a study on the assessment of the surveillance program as well as the effectiveness of the enforced control measures. More studies on vaccination efficacy and vaccination implementation strategies should be conducted to support a robust surveillance system. The findings from this study might be useful for both the livestock authority and various stakeholders in their goal to develop an enhanced control strategy and surveillance system that could enable them to control and eradicate FMD throughout the country.

## Figures and Tables

**Table 1:** Poisson regression model for foot and mouth disease outbreaks in Thailand from 2008 to 2019.

Period	Estimate	Standard Error	Z-Value	*p*-Value
Period * 1	Reference class			
Period 2	0.526	0.140	3.75	<0.001

* Period 1 = 2008–2013, Period 2 = 2014–2019.
